# New Species of Ascomycetes from Two Hypersaline Endorheic Lagoon Complexes in Zaragoza Province (Aragon Community, Spain)

**DOI:** 10.3390/jof11020139

**Published:** 2025-02-12

**Authors:** María Barnés-Guirado, José F. Cano-Lira, Andrew N. Miller, Alberto M. Stchigel

**Affiliations:** 1 Mycology Unit, Medical School, University Rovira i Virgili, C/Sant Llorenç 21, 43201 Tarragona, Spain; maria.barnes@estudiants.urv.cat (M.B.-G.); albertomiguel.stchigel@urv.cat (A.M.S.); 2 Illinois Natural History Survey, University of Illinois Urbana-Champaign, 1816 South Oak Street, Champaign, IL 61820-6970, USA; amiller7@illinois.edu

**Keywords:** ascomycota, basins, biodiversity, extremophiles, fungi, salt tolerant

## Abstract

Although certain hypersaline endorheic lagoons in Spain have been investigated to evaluate the composition, spatial structure, and ecological roles of their macro- and microbiota, the fungi inhabiting these niches remain largely unexplored. In this study, we isolated several microfungi from the *Salada Grande de Chiprana* and *La Playa lagoons*, located in the *Saladas de Chiprana* (Directed Natural Reserve and Ramsar Wetland) and the *Saladas de Sástago–Bujaraloz* (Ramsar Wetland), respectively. As a result of morphological characterization and phylogenetic analysis using four informative molecular markers, we report the discovery of two new species of the genus *Montagnula* (*M.*), *M. globospora* and *M. terricola*, as well as one new species of *Monosporascus* (*Mo.*), *Mo. auratispora*. *Montagnula globospora*, isolated from a sediment sample from *Salada Grande de Chiprana lagoon*, is the only species of the genus producing unicellular, globose ascospores inside cleistothecial ascomata with a cephalothecoid peridium. *Montagnula terricola* was originally isolated from a soil sample in Malawi (ex-type strain). However, we have also identified another strain of this species from a sediment sample collected at *La Playa lagoon*. The remarkable features of *M. terricola* are the production of non-cephalothecoid cleistothecial ascomata and bicellular, bi-cupulate ascospores. Regarding *Mo. auratispora*, it was isolated from sediments of *Salada Grande de Chiprana* and is characterized by the production of golden-brown ascospores that do not turn black with age. Also, due to the results of our phylogenetic analysis, we transferred *Herpotrichia striatispora* to the genus *Montagnula*, as *M. striatispora*, and *Montangula jonessi* to the new genus *Neomontagnula* (*N.*), as *N. jonessi*.

## 1. Introduction

Hypersaline endorheic lagoons are located within endorheic basins—closed systems with no direct connection to seas, oceans, or rivers—that are predominantly found in salt flats or depressions within arid or semi-arid continental regions [1,2]. These lagoons represent fragile ecosystems that are particularly sensitive to fluctuations in air temperature and precipitation patterns. Consequently, they are highly vulnerable to the impacts of global climate change, as well as to direct anthropogenic pressures [3,4]. Furthermore, these lagoons support a rich biodiversity that differs from that of permanent lagoons and lakes, often contributing more significantly to regional biodiversity than larger water bodies and providing a refuge for uncommon and rare species [5,6].

The *Saladas de Chiprana* and the *Saladas de Sástago-Bujaraloz* are endorheic lagoon complexes located in the Ebro Basin (Zaragoza Province, Northeast Spain). Since 1994, the *Saladas de Chiprana* have been protected under the Ramsar convention and have been regularly monitored to preserve their unique characteristics [4,7]. The *Salada Grande de Chiprana* (41°14′26.8″ N 0°10′59.1″ W) is the main and deepest lagoon in the complex, and it is the only known permanent hypersaline lagoon in Western Europe [8]. The local climate is semi-arid Mediterranean, characterized by intense summer droughts and increased winter precipitations [9]. This hypersaline lagoon has a surface area of approximately 31.5 ha and a variable depth between 3.5 m and 5.6 m, and it is situated at 137 m a.s.l. The mean annual temperature is approximately 15.5 °C, with minimal and maximal temperatures of 3 °C and 34 °C, respectively [10].

The other lagoon complex, the *Saladas de Sástago-Bujaraloz*, is located on a platform of ~400 m a.s.l. and comprises more than a hundred basins. It is one of the best-preserved and the most representative lagoon systems associated with these basins and, thus, is protected by the Ramsar Convention on Wetlands [7,11]. The largest of these lagoons is the *Laguna de La Playa*, situated approximately 20 km south of the *Salada Grande de Chiprana*. It is the largest salt flat in the complex, with an area of 239.9 ha and a length of 2.7 km. *Laguna de La Playa* has one of the highest occurrences of water presence, following *La Salineta*, with water present on 77% of the 52 dates studied between 1984 and 2004. The largest water surface area recorded was 187 ha in January 1987, and the maximum depth of 51 cm was measured in December 1994 [7,12]. This lagoon complex shares its local climate with the *Saladas de Chiprana* [11,13]. However, unlike the *Salada Grande de Chiprana, La Playa lagoon* suffers dramatic changes in surface area and salt concentration throughout the year and over time, as can be observed by the variation of different physical–chemical parameters in the comparative Table 1.

Despite the archaeal and bacterial communities of some hypersaline lagoons being well-characterized [14,15,16,17,18,19], the contribution of halophilic and salt-tolerant fungi to these microbiotas has only recently been acknowledged. Species of “black yeasts”, such as *Hortaea werneckii* and *Trimmatostroma salinum*, are present in marine-origin solar salterns [20,21,22,23,24], where they are well-adapted to proliferate in water and sediments with high salt concentrations [25,26,27]. The most halophilic of these fungi, *Wallemia ichthyophaga*, can grow in NaCl concentrations up to 250 g/L [28]. Many other genera of filamentous fungi and yeasts have been found in salterns and natural salt lakes, such as the Dead Sea [20,29]. Recently, we described the new fungus *Dactyliodendromyces holomorphus*, from the waters of *Laguna de Pito*, a hypersaline endorheic lagoon in the *Saladas de Sástago-Bujaraloz* complex [30]. However, the fungus recovered in this place proved to be only moderately salt tolerant to non-salt tolerant.

Although extreme environments are recognized as reservoirs of a diverse array of fungi with biotechnological potential [31,32,33], research on fungi in hypersaline lagoons remains limited due to the underexplored nature of these ecosystems. Nevertheless, Gomoiu et al. [34] reported the production of hydrolytic enzymes by fungi isolated from brackish and hypersaline lakes, while Georgieva et al. [35] evaluated the antimicrobial potential of halo-alkali-tolerant fungi from the Big Tambukan Saline Lake (Northern Caucasus). Additionally, several halophilic fungal species from different environments have been reported to exhibit antibacterial activity [32,36,37,38].

Given the absence of prior studies on fungal diversity in the *Salada Grande de Chiprana* and *La Playa lagoons*, our study aimed to improve the understanding of the fungal biodiversity inhabiting these endorheic hypersaline lagoons. To achieve this, we employed culture-dependent techniques combined with a polyphasic taxonomic approach to identify fungal species and determine their phylogenetic position and relationships with other fungi.

**Table 1:** Main physical-chemical parameters of the *Salada Grande* and *La Playa lagoons*.

Parameter	*Salada Grande* (*Saladas de Chiprana Lagoon* Complex) [14]	*La Playa* (*Saladas de Sástago-Bujaraloz Lagoon* Complex)
pH	9.2, 9.6	8.0 [15], 6.75–8.3 [39], 8.2 [40]
Temperature (°C; at surface)	8.85, 11.4	23.8 [15], 16.4 [40]
Salinity (g/L)	70–80	18.38 [40]
Conductivity (mS/cm)	46, 40	>200 [15], 28.8 [40]
Dissolved O_2_ (mg/L)	8.65, 7.86	9.4 [15], 9.1 [40]
Phosphates (ppb)	ND	<100 [15]
Nitrates (ppm)	ND	<1 [15], <2 [40]
Ammonia (ppm)	2.5	0.6 [15]
Cl^−^ (ppm)	8214	27,050–166,371 [39]
SO_4_^2−^ (ppm)	25,679	8309–94,955 [39]
Na^+^ (ppm)	9619	19,237–120,657 [39]
K^+^ (ppm)	192	605–16,750 [39]
Ca^2+^ (ppm)	586	84–968 [39]
Mg^2+^ (ppm)	8233	3037–25,235 [39]

## 2. Materials and Methods

### 2.1. Characterization of the Studied Area

Different physical–chemical parameters of the *Salada Grande* and *La Playa lagoons*, available in the literature, were compiled in Table 1.

### 2.2. Sampling and Fungal Isolation

**Figure 1:**
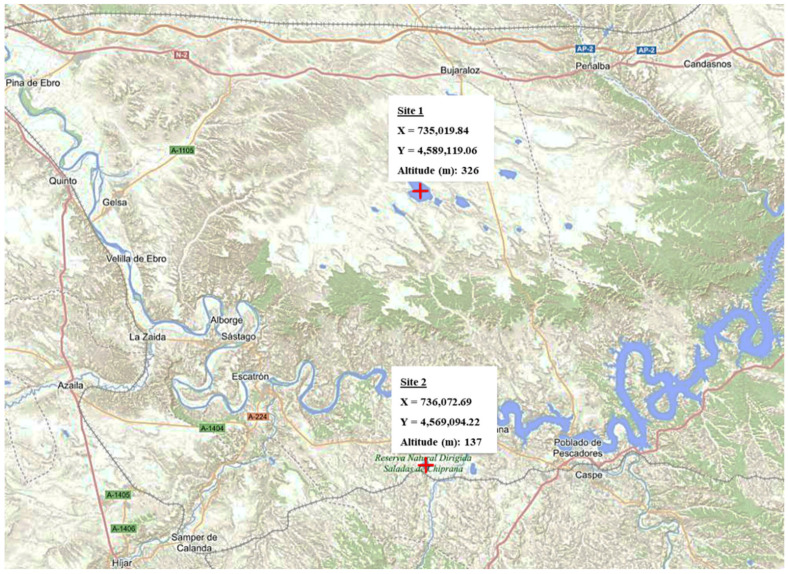
Sampled locations (https://www.ign.es/iberpix/visor; accessed on 19 March 2024).

We collected several samples of the hypersaline waters (from superficial and intermediate [from 10 to 50 cm depth depending of the lagoons] layers), lagoon sediments (of up to 60 mm depth), and soils (A horizon, up to 50 mm deep) surrounding the *La Playa* (Figure 1, Site 1) and *Salada Grande de Chiprana lagoons* (Figure 1, Site 2) in May 2021 and July 2022, respectively. To collect water and sediment samples, sterile plastic containers for biological samples, held with an extendable and retractable narrow telescopic grabber arm rod, were introduced into the aquatic medium and subsequently sealed with their respective screw caps.

The salinity and pH of the water samples were measured by an Aokuy refractometer (Shenzhenshi Jinshenghe Shangmao Youxiangongsi, Guangdong, China) and SRSE water test strips (Tepcom GmbH & Co. KG, Bendorf, Germany). The samples were collected using 100 mL sterile plastic containers and transported to the laboratory to be refrigerated at 4–7 °C. In order to isolate the largest possible number of fungal taxa, the following culture media were used: 2% malt extract agar (MEA; Difco Inc., Detroit, MI, USA; [41], supplemented with 30% glycerol); potato–dextrose agar (PDA; Laboratorios Conda S.A., Madrid, Spain; [42] plus 10% NaCl); ascospore agar (AA, 5 g potassium acetate, 1.25 g yeast extract, 0.5 g dextrose, 15 g agar-agar, 500 mL distilled water; [43]), and 18% of glycerol agar (G18; 2.5 g peptone, 5 g dextrose, 0.5 g KH_2_PO_4_, 0.25 g MgSO_4_, 90 mL glycerol, 7.5 g agar-agar, 410 mL distilled water; [44]). Bacterial growth was prevented by adding 250 mg/L of L-chloramphenicol to each culture media previously sterilized in an autoclave. For each sample of water, 5, 15, and 30 mL were filtered through a filter membrane with a 0.45 µm pore diam. (Millipore SA, Molsheim, France) using a vacuum pump. Then, the filter membranes were aseptically placed (using sterilizable metal tweezers) onto the culture media mentioned previously and deposited into 90 mm diam. sterile disposable Petri dishes. Samples of sediment were vigorously shaken inside their original containers and then settled for one minute for sedimentation. Then, the upper water layer was removed, and the solid sediment was dried over several layers of sterile filter paper placed on plastic trays [45]. One gram of dried sediment and an equal amount of soil sample were evenly sprinkled onto each culture media in 90 mm diam. sterile disposable Petri dishes. Additionally, to break the dormancy of the fungal spores, sediment samples were treated with 5% acetic acid [46,47]. Every sample was cultured in duplicate and incubated in darkness at 15 °C and 37 °C. Petri dishes were examined daily using a stereomicroscope for up to two months to detect fungal development. After visualization, every single colony was transferred to a 55 mm Petri dish containing oatmeal agar (OA; 15 g of filtered oat flakes, 7.5 g agar, 500 mL tap water; [41]) using sterile disposable tuberculin-type needles and syringes and then incubated at room temperature until obtaining pure cultures. Fungal strains were deposited in the culture collection of the Faculty of Medicine of Reus (FMR; Tarragona Province, Spain), and uncommon taxa, as well as the ex-type strains and the herborized specimens (holotypes), were deposited at the Westerdijk Fungal Biodiversity Institute (CBS; Utrecht, The Netherlands) for their preservation.

### 2.3. Phenotypic Study

The macroscopic characterization of the colonies was carried out on OA, MEA, PDA, and potato–carrot agar (PCA; 10 g potato, 10 g carrot, 6.5 g agar, 500 mL distilled water) after incubation for 14 days at 25 °C in darkness [41,42]). The color description of the colonies was made according to Kornerup and Wanscher [48]. Cardinal temperatures of growing were determined on PDA, ranging from 5 to 40 °C at 5 °C intervals, with an additional measurement at 37 °C. Microscopic characterization of vegetative and reproductive structures was carried out by visualizing the vegetative and reproductive fungal structures from the colonies grown on OA in the previously stated conditions. At least 30 measurements of each structure were taken from slide mountings using Shear’s medium (3 g potassium acetate, 60 mL glycerol, 90 mL ethanol 95%, 150 mL distilled water; [49]) using an Olympus BH-2 bright-field microscope (Olympus Corporation, Tokyo, Japan). Micrographs were taken using a Zeiss Axio-Imager M1 light microscope (Zeiss, Ober-kochen, Germany) with a DeltaPix Infinity × digital camera using Nomarski differential interference contrast.

### 2.4. DNA Extraction, Amplification, and Sequencing

Total genomic DNA was extracted from colonies grown on PDA for 7 to 10 days at 25 °C in darkness following the modified protocol of Müller et al. [50] and quantified by a Nanodrop 2000 (Thermo Scientific, Madrid, Spain). The molecular markers used for each fungal strain were selected and amplified according to the bibliography. The primers used to amplify the internal transcribed spacers (ITS) region and the D1-D2 domains of the 28S nrRNA (LSU) were ITS5/ITS4 [51] and LR0R/LR5 [52], respectively. The primers used to amplify fragments of the beta-tubulin gene (*tub*2) were BT2a/BT2b [53]. For the fragments of the translation elongation factor 1α (*TEF*-1α), the primer pair EF-728F/2218R [54,55] was used. Single-band PCR products were stored at −20 °C and sequenced at Macrogen Europe (Macrogen Inc., Madrid, Spain) with the same primers employed for amplification. The software SeqMan v. 7.0.0 (DNAStarLasergene, Madison, WI, USA) was to used edit and assemble the consensus sequences.

### 2.5. Phylogenetic Analysis

The consensus sequences were compared with all of the sequences available at the National Center for Biotechnology Information (NCBI) database using the Basic Local Alignment Search Tool (BLAST; https://blast.ncbi.nlm.nih.gov/Blast.cgi, accessed on 3 January 2025). A maximum level of identity (MLI) of ≥ 98% was settled for species-level identification [56]. To perform single and combined phylogenetic analysis of all the molecular markers required for each genus, we performed a sequence alignment with the software MEGA (Molecular Evolutionary Genetics Analysis) v. 7.0. [57], using the ClustalW algorithm [58] and refining with MUSCLE [59] or/and manually, if necessary. Afterward, the phylogenetic reconstruction was performed by two different methods, including the maximum likelihood (ML), carried out with the software RAxML-HPC2 on XSEDE v. 8.2.12 [60] from the CIPRES Science gateway portal [61], and the Bayesian inference (BI), performed by using MrBayes v.3.2.6 [62]. The best substitution model for all of the gene matrices was established by the CIPRES Science gateway portal (ML) and by the software jModelTest v.2.1.3 following the Akaike criterion (BI) [63]. Regarding the ML analysis, the phylogenetic support for internal branches was established by 1000 ML bootstrapped pseudoreplicates, only considering significant bootstrap support (BS) values ≥70 [64]. Regarding the BI analysis, 5 million Markov Chain Monte Carlo (MCMC) generations were used, with four runs (one cold chain and three heated chains), sampling every 1000 generations. Before calculating the 50% majority-rule consensus tree and posterior probability values (PP), the first 25% of samples were discharged. Only pp values ≥0.95 were considered significant [65]. FigTree v.1.3.1 (http://tree.bio.ed.ac.uk/software/figtree/, accessed on 3 January 2025) was used to plot the resulting phylogenetic trees. The DNA sequences generated in this study were deposited in GenBank (Table 2). The phylogenetic trees generated in this study and their associated alignments were deposited in Zenodo (https://zenodo.org, accessed on 3 January 2025).

## 3. Results

### 3.1. Salinity and pH of the Water Samples

Water samples displayed a pH of 8.2 and a salinity of 34‰ *w*/*v* for the *La Playa* and 8.4 and 62 ‰ *w*/*v* for the *Salada Grande de Chiprana lagoons*.

### 3.2. Phylogeny

Among all the strains isolated during the development of this study, the strains FMR 19580, FMR 20550, and FMR 20333 grabbed our attention due to their unique morphology, leading to an in-depth morphological and phylogenetic analysis. The results of the BLAST search using ITS, LSU, *tub*2, and *TEF*-1α sequences for these three strains are summarized in Table 3.

Based on the BLAST results, we conducted two phylogenetic analyses: one including species from the family *Didymosphaeriaceae*, given the preliminary placement of our strains FMR 19580 and FMR 20550 within the genus *Montagnula*, which belongs to this family, and another including species from the genus *Monosporascus*, based on the preliminary placement of our strain FMR 20333 within this genus.

**Figure 2:**
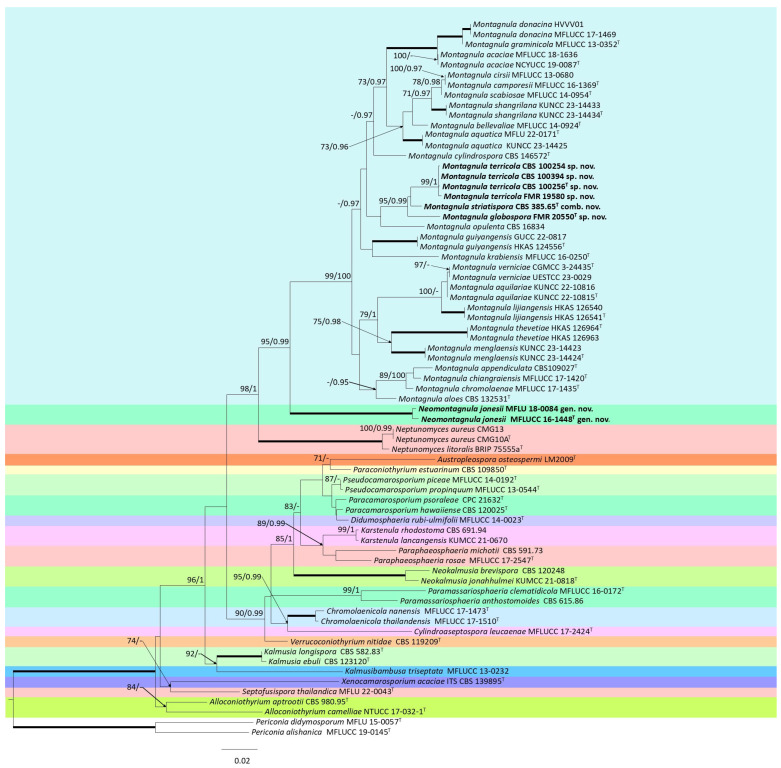
Phylogenetic analysis of members of the family *Didymosphaeriaceae* was conducted using ITS, LSU, and *TEF*-1α nucleotide sequences. RA × ML bootstrap support (BS) values ≥70% and Bayesian posterior probabilities (PP) ≥0.95 are shown above the branches. Branches with 100% BS/1 PP are indicated as broad lines. Novel genus, species, and combinations are indicated in bold type. The tree is rooted to *Periconia didymosporum* MFLU 15-0057 and *Periconia alishanica* MFLUCC 19-0145. ^T^ = Ex-type strains.

For the individual analysis containing species belonging to the *Didymosphaeriaceae* family, the individual dataset for ITS, LSU, and *TEF*-1α did not display any conflicts related to the tree topologies for the 70% reciprocal bootstrap trees. Therefore, a multi-gene analysis was performed. The final concatenated dataset included 69 ingroup strains belonging to the genera *Alloconiothyrium*, *Austropleospora*, *Chromolaenicola*, *Cylindroaseptospora*, *Didymosphaeria*, *Kalmusia*, *Kalmusibambusa*, *Karstenula*, *Montagnula*, *Neokalmusia*, *Neptunomyces*, *Paracamarosporium*, *Paraconiothyrium*, *Paramassariosphaeria*, *Paraphaeosphaeria*, *Pseudocamarosporium*, *Septofusispora*, *Verrucoconiothyrium,* and *Xenocamarosporium*, plus two outgroup strains of the genus *Periconia*. The alignment encompassed a total of 2179 characters including gaps (484 for ITS, 814 for LSU, and 881 for *TEF*-1α), 466 of them parsimony informative (193 for ITS, 106 for LSU, and 167 for *TEF*-1α) and 626 of them variable sites (226 for ITS, 175 for LSU, and 225 for *TEF*-1α). The tree obtained by the BI analysis was congruent and similar in topology to the one obtained through the ML analysis (Figure 2). Regarding the ML analysis, K2 + G + I was the best-fitting model for ITS and LSU and TN93 + G + I was the best-fitting model for *TEF-1α*. Regarding the BI analysis, SYM + I + G was the best-fitting model for ITS, and GTR + I + G was the best-fitting model for both LSU and *TEF*-1α. The support values showed slight differences between the two analysis methods. However, no incongruences were shown.

The phylogenetic analyses revealed seven fully supported clades formed by different family members of *Didymosphaeriaceae*. Most of the species of the *Montagnula* genus clustered in a main clade (99% BS, 1 PP), whereas *Montagnula jonesii* was placed alone in a distant, independent, and fully supported terminal clade. Consequently, *Montagnula jonesii* was considered a novel genus. Within the *Montagnula* clade, our strains were placed in a well-supported terminal clade (95% BS, 0.99 PP), the strain FMR 19580 clustered in the same terminal branch (99% BS, 1 PP) with CBS 100254, CBS 100256, and CBS 100394 and FMR 20550 were placed in an independent well-supported branch. The strains CBS 100254, CBS 100256, and CBS 100394 were deposited in the Westerdijk Fungal Biodiversity Institute collection under the non-validated name of ‘*Aporospora terricola*’. Our phylogenetic analysis demonstrates that these strains are co-specific to our strain FMR 19580, representing a novel species of the genus *Montagnula*. On the other hand, the strain CBS 385.65, previously classified within the genus *Herpotrichia* as the type strain of *H. striatispora*, was included in our phylogenetic analysis because the BLAST searches using all of the phylogenetic markers placed it within the genus *Montagnula*. Since CBS 385.65 was positioned as an independent species in the same terminal clade as the ‘*Aporospora terricola*’ strains (including FMR 19580 and FMR 20550), it represents a novel combination, *Montagnula striatispora*.

**Figure 3:**
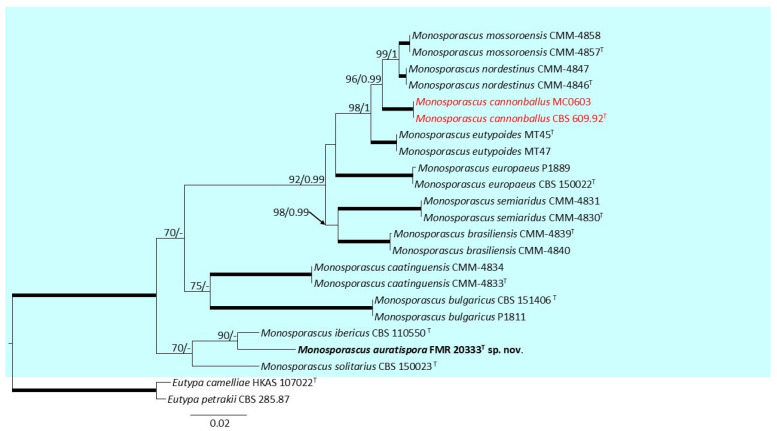
Phylogenetic analysis of members of *Monosporascus* using the molecular markers ITS, LSU, *tub2,* and *TEF*-1α. RA × ML bootstrap support (BS) values ≥70% and Bayesian posterior probabilities (PP) ≥0.95 are displayed above the branches. Branches with 100% BS/1 PP are indicated as broad lines. Novel species is indicated in bold; type species in red. Tree rooted to *Eutypa camelliae* HKAS 107022 and *Eutypa petrakii* CBS 285.87. ^T^ = Ex-type strain.

Regarding the individual analysis containing species belonging to the genus *Monosporascus* (Figure 3), to carry out a multi-gene analysis, we first corroborated that the tree topologies for the 70% reciprocal bootstrap trees of the individual molecular markers, ITS, LSU, *tub2,* and *TEF*-1α did not show contradictions. The final multi-gene analysis dataset included 21 ingroup strains of the genus *Monosporacus*, and, as outgroups, *Eutypa petrakii* CBS 285.87 and *Eutypa camelliea* HKAS 107022. The alignment comprised 2425 characters, including gaps (535 for ITS, 688 for LSU, 553 for *tub2,* and 649 for *TEF*-1α). Among the total characters, 422 of them were parsimony informative (122 for ITS, 39 for LSU, 90 for *tub2,* and 171 for *TEF*-1α) and 506 of them were variable sites (133 for ITS, 54 for LSU, 120 for *tub2,* and 199 for *TEF*-1α). For both the ML and the BI analyses, the support values obtained displayed little to no differences. Thus, both analyses were consistent. In the ML analysis, the model that fitted the best for all of the molecular markers was K2 + G. In the BI analysis, the model that fitted the best for ITS, *tub2,* and *TEF*-1α was K80 + G, and the model that fitted the best for LSU was K80 + I.

The phylogenetic analysis revealed a fully supported main clade encompassing all species of *Monosporascus*, which was further divided into three subclades. The first of these subclades is well-supported (92 BS, 0.99 PP) and includes most species within the genus. The second subclade comprises two fully supported terminal branches corresponding to the species *Monosporascus caatinguensis* and *Monosporascus bulgaricus*. The third subclade contains two terminal branches, one with *Monosporascus solitarius* and the other with *Monosporascus ibericus* and our strain FMR 20333, with enough phylogenetic distance between them to be considered separate species.

### 3.3. Taxonomy

*Pleosporales* Luttr. ex M.E. Barr, Prodr. Cl. Loculoasc. (Amherst): 67 (1987). Mycobank MB 90563.

*Didymosphaeriaceae* Munk, Dansk bot. Ark. 15 (no. 2): 128 (1953). Mycobank MB 80702.

*Montagnula* Berl., Icon. fung. (Abellini) 2 (2–3): 68 (1896). Mycobank MB 3265.

*Type species*—*Montagnula infernalis* (Niessl) Berl. 1896 (designated by Clements and Shear, Gen. fung., Edn 2 (Minneapolis): 276. 1931).

**Figure 4:**
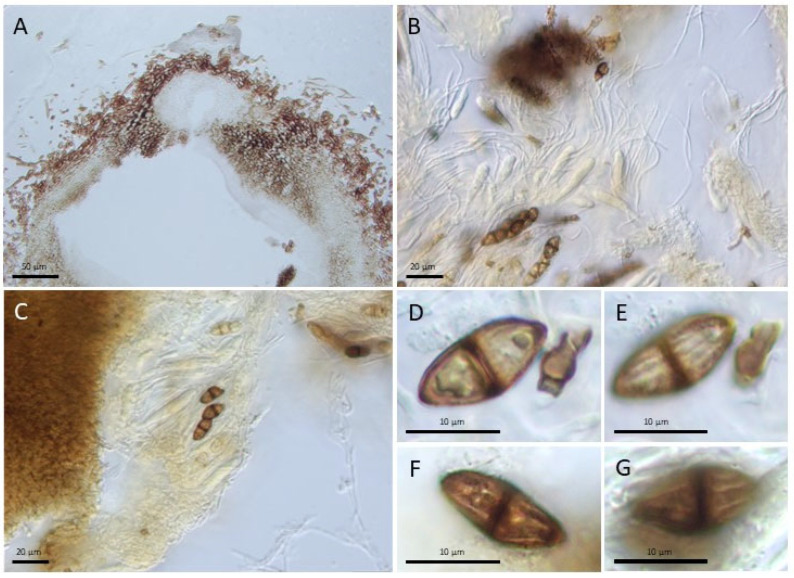
*Montagnula striatispora* ILL00000264 (isotype of Papendorf 83). (**A**). Ascoma, cross-section. (**B**,**C**). Hymenium, showing young and mature asci within ascospores and paraphyses. (**D**–**G**). Mature, superficially striated ascospores. Scale bars: (**A**) = 50 µm; (**B**,**C**) = 20 µm; (**D**–**G**) = 10 µm.

*Montagnula striatispora* (Papendorf and Arx, 1967) Barnés-Guirado, Stchigel and Cano, comb. nov. MycoBank MB854493 Figure 4.

Basionym—*Herpotrichia striatispora* Papendorf and Arx, Nova Hedwigia 12 (3 + 4): 395 (1967).

*Description*: Papendorf and Arx (1967).

*Notes*: The distinctive features of *M. striatispora* are related to the nature of their ascospores, which are bicellular, asymmetrically biconical with acute ends, and longitudinally striate. Also, the hamathecium is composed of paraphyses, not as in the most of species of the genus (pseudoparaphysate), having a peryphysate ostiolate ascomata.

**Figure 5:**
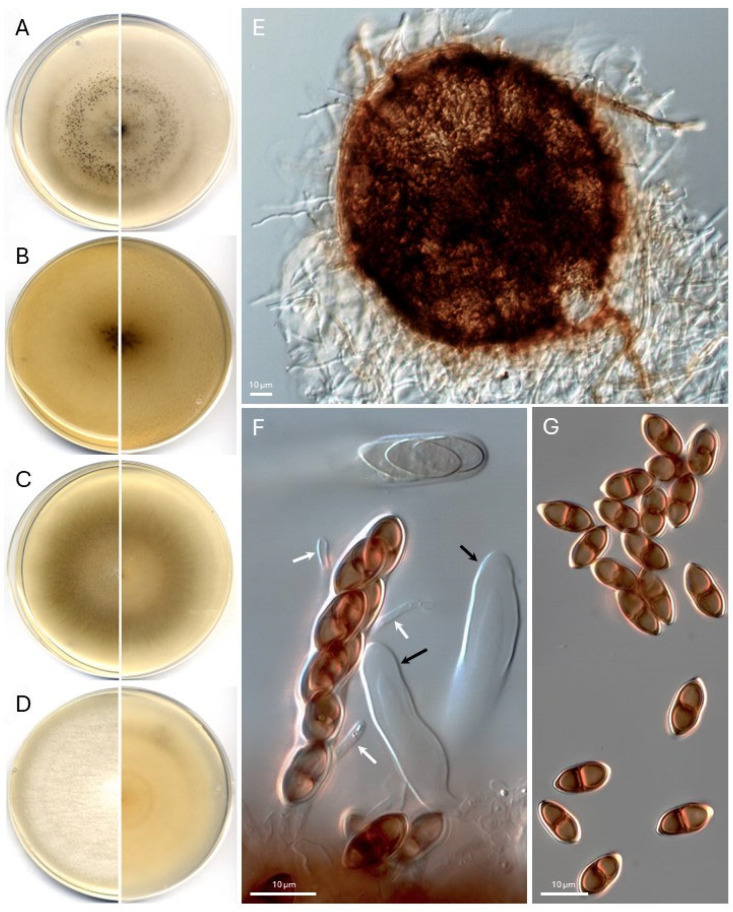
*Montagnula terricola* CBS 100256^T^. (**A**–**D**). Colonies on PCA, OA, MEA, and PDA, respectively, after two weeks at 25 ± 1 °C (left, surface; right, reverse). (**E**). Ascoma. (**F**). Bitunicate asci (young, black arrows) and paraphyses (white arrows). (**G**). Mature ascospores. Scale bars: (**E**–**G**) = 10 µm.

*Montagnula terricola* Barnés-Guirado, Cano and Stchigel, sp. nov. MycoBank MB850890 Figure 5.

*Etymology*. From Latin terra-, soil, and *-colere*, living in, because of the (mostly) geophilic habit of the fungus.

*Description: Mycelium* superficial to immersed, composed of hyaline to brown, septate, smooth-walled to rugose (due to the external deposition of melanin-like pigments), thin- to thick-walled hyphae 2–9 µm wide, often anastomosing and grouped to form rope-like dark-brown structures. *Sexual state* — *Ascomata* erumpent, dark brown, non-ostiolate, setose, globose to subglobose, 130–260 µm diam.; *peridial wall* brown to dark brown, translucent, 2–3-layered, 5–8 µm thick, of textura angularis in upper view, composed by flattened, brown polygonal cells of 4–12 µm diam., covered by an irregular net of dark brown, septate, smooth-walled to tuberculate anastomosing hyphae; *setae* scarce, distributed irregularly on the surface of the ascomata, septate, brown at the base but becoming subhyaline to hyaline at the rounded apex, sinuous, up to 7 µm wide at the base, up to 100 µm long, and covered by a gelatinous sheath then becoming tuberculate with the age. *Asci* 8-spored, bitunicate, cylindric-clavate, 38–48 × 4.5–7.5 µm, non-stipitate, without apical apparatuses. *Paraphyses* hyaline, septate, unbranched, 40–55 × 2 µm. *Ascospores* are two-celled, non-constricted to slightly constricted at the broad median septum, smooth- and thick-walled, chocolate brown when mature, broadly fusiform to biconical or bicampanulate, 9.5–12 × 5 µm, without appendages nor gelatinous sheath; aberrant ascospores seen in culture, one-celled, brown, smooth- to rough-walled, globose to ellipsoidal, and 5–7 µm diam. when globose, similar in size to the “normal” ones when ellipsoidal. *Asexual state*—not observed.

*Culture characteristics* (14 days at 25 °C)—Colonies on PDA reach 83 mm diam., slightly raised at center flattened at the edges, cottony, smooth, white (1A1) at center, grey (4B1) towards periphery, filamentous margins, sporulation absent; reverse yellowish orange (4A6) to pale yellow (4A3) at center, greyish yellow (4B3) to pale yellow (4A3) at periphery, and diffusible pigment absent. Colonies on PCA reach 82 mm diam., slightly raised at center flattened at the edges, cottony to velvety, smooth, white (1A1) to greyish brown (5E3) at center, olive brown (4F8) to grey (4B1) towards periphery, filamentous margins, sporulation moderate to abundant, reverse, yellowish-brown (5F8) at center, olive brown (4F3) to grey (4B1) at towards periphery, and diffusible pigment absent. Colonies on OA reach 80 mm diam., flattened and immersed hyphae towards periphery, cottony to velvety, smooth, white (1A1) to olive brown (4F3) at center, olive brown (4E4) to grey (4B1) at the edges, entire, sporulation moderate; reverse brownish grey (4F2) at center, olive brown (4E4) to grey (4B1) at the edges, and diffusible pigment absent. Colonies on MEA reach 75 mm diam., slightly raised at center and flattened at the edges, cottony to velvety, smooth, white (1A1) at center, grey (4B1) to brownish grey (4D2) towards periphery, filamentous margins, sporulation absent; reverse yellowish orange (4A6) at center, olive brown (4D3) to grey (4B1) at the edges, and diffusible pigment absent. Cardinal growth temperatures: minimum of 15 °C, an optimum of 30 °C, and a maximum of 40 °C.

*Specimen*: MALAWI, Southern Region Mulanje Dist., Mulanje Mountain Chamble Basin, track to Knife Edge, 15°55′ S 35°34′ E, 2100 m.a.s.l., isolated from soil, 13/04/1991, collected and isolated by J.C. Krug, holotype TRTC 51988, and cultures ex-type CBS 100256 = ATCC 201452 = FMR 19807.

*Other specimens*: TRTC 51987 = CBS 100254 = ATCC 201451 = FMR 19806, Egypt, Western Desert, Wadid Gedeed Governorate, Dakleh Oasis, Ein Birbiyeh, 25°32′ N 29°19′ E, isolated from soil near an irrigation canal, collected and isolated by J.C. Krug; TRTC 51989 = CBS 100394 = ATCC 201650 = FMR 19805, N-exposed cliff, Tunisia, 5 km NW of Ksar Haddada, NW from Tatahouine 470 m.a.s.l., soil attached to rhizoids of *Grimmia orbicularis*, collected and isolated by J.C. Krug; CBS 150901 = FMR 19580, Spain, Aragon Community, Zaragoza province, La Playa lagoon, 41°25′16.2″ N 0°11′18.4″ W, isolated from lagoon sediment, 14/05/2021, collected by María Barnés, Alan O. Granados and José F. Cano, and isolated by María Barnés.

*Notes*: All four strains of *Montagnula terricola* produce globose to subglobose (150–230 µm diam.), dark brown, setose, thin-walled, non-ostiolate ascomata, non-stipitate cylindric-clavate (43–50 × 6–10 µm) asci, and smooth-walled ascospores, whereas *Montagnula striatispora* produces a globose to pear-shaped (340–600 µm diam.), black, glabrous, very thick-walled, ostiolate ascomata, clavate (62–90 × 9–12 µm) asci, and ascospores ornamented with longitudinal ridges. However, it is remarkable that both species produce true paraphyses, not as was described for most of the species of the genus, which produce pseudoparaphyses.

**Figure 6:**
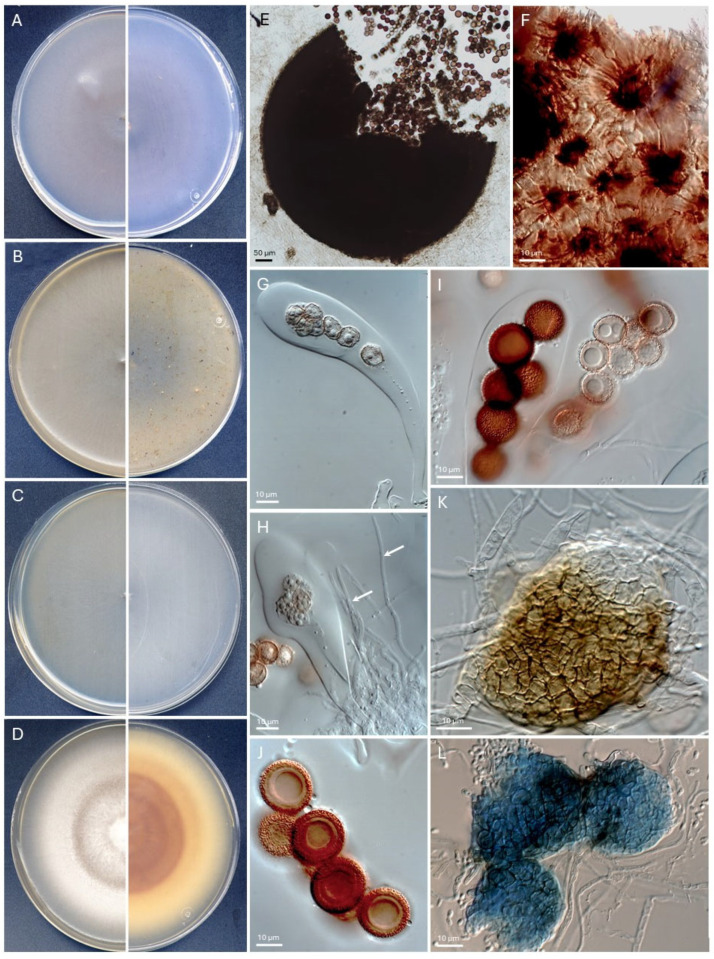
*Montagnula globospora* CBS 152803^T^. (**A**–**D**). Colonies on PCA, OA, MEA, and PDA, respectively, after two weeks at 25 ± 1 °C (left, surface; right, reverse). (**E**). Broken ascoma. (**F**). Detail of the cephalothecoid peridium. (**G**,**H**). Young asci and paraphysis (white arrows). (**I**). Mature asci within ascospores. (**J**). Mature ascospores, most in cross-section. (**K**). Pycnidium. (**L**). Mature pycnidia (Cotton Blue stained). Scale bars: (**E**) = 50 µm; (**F**–**L**) = 10 µm.

*Montagnula globospora* Barnés-Guirado, Cano and Stchigel, sp. nov. MycoBank. MB 854553 Figure 6.

*Etymology* From the Latin globosus-, globose, and -sporae, spore, because of the nature of their ascospores.

*Description*: Mycelium superficial composed of hyphae hyaline to subhyaline, septate, thick-walled, branched, 1.0–2.5 µm wide. *Sexual state*—*Ascomata* cleistothecial, dark brown to black, subglobose, 220–385 × 230–395 µm; *peridium* 5–15 µm thick, outer wall 1–5-layered, composed of pale golden brown, smooth- and thick-walled, translucent prismatic cells, 5–15 µm in the main axis, inner wall 1–4-layered, composed of dark brown, smooth- and thick-walled, slightly translucent flattened prismatic cells, 5–15 µm in the main axis, cephalothecoid, composed of dark brown polygonal plates of 25–60 µm diam., separating by preformed paler sutures dark brown to black, and covered by subhyaline septate hyphae. *Paraphyses hyaline* is septate, unbranched, 45–55 × 1–1.5 µm. *Asci* bitunicate, 5 to 8-spored, ascospores uni- to biseriate, cylindric clavate to clavate, 105–135 × 24–34 µm, and without apical apparatuses. *Ascospores* one-celled, dark brown, very thick-walled, granulate, globose to subglobose, 9.5–19.5 × 9.5–17 µm, and guttulate. *Asexual state*—*Conidiomata* pycnidial, mostly unilocular, occasionally bilocular, clustering or more rarely solitary, pale brown to golden brown, becoming paler towards the apex, globose, broadly ellipsoidal, rarely lenticular, 20–40 µm diam, opening by a late dehiscence at the upper part of the peridium; *peridium* translucent, with a few digitiform projections, 1–2-layered, 4–7 µm thick, textura angularis, composed of smooth- and thin-walled, flattened polygonal cells up to 5 µm diam.; *conidiogenous cells* phialidic, globose, 1–1.5 × 2–3 µm, lacking a distinctive collarette or neck; *conidia* enteroblastic, unicellular, solitary, hyaline, cylindric, 2.5–4 × 1–2 µm, and produced in mass into a hyaline mucilaginous matrix.

*Culture characteristics* (after 14 d at 25 °C)—Colonies on PDA reaching 85 mm diam., raised at the center, flattened at the margins, cottony to velvety, radially sulcate at the center, smooth at the margins, white (1A1) to yellowish brown (5E8) at the center, white (1A1) to light brown (5D6) at the filamentous margins, sporulation abundant; reverse brown (7E8) at center, golden brown (5D7) at the margins; soluble pigment absent. Colonies on PCA reaching 85 mm diam., raised at the center, flattened at the entire margins, velvety, smooth, pale red (12A3), sporulation abundant; reverse reddish-white (12A2); and soluble pigment absent. Colonies on the OA reach 85 mm diam., flattened velvety, smooth, hyaline, margins filamentous, sporulation abundant; reverse hyaline; and soluble pigment absent. Colonies on MEA reach 82 mm diam., flat, velvety, smooth, hyaline, margins filamentous, sporulation abundant; reverse hyaline; and soluble pigment absent. Cardinal temperatures of growth: minimum of 15 °C, an optimum of 30 °C, and a maximum of 37 °C.

*Specimen*: FMR 20550 = CBS 152803. SPAIN, Aragon community, Zaragoza province, *Salada Grande de Chiprana*, 41°14′19.2″ N 0°10′49.6″ W, isolated from lagoon sediment, 11 July 2022, collected by María Barnés, Alan O. Granados and José F. Cano, and isolated by María Barnés, holotype CBS H-25643.

*Notes*, *Montagnula globospora* differs from all previously known species by the production of cleistothecioid ascomata with a cephalothecoid peridium, one-celled, granulate ascospores, and by the presence of a pycnidial asexual state. Also, in addition to *M. terricola* and *M. stratispora*, *M globospora* forms true paraphyses.

Because the description of the genus *Montagnula* does not include species with cleistothecial ascomata nor unicellular ascospores, thus, we emended the generic description as follows:

*Montagnula* Berl. emended by Barnés-Guirado, Cano, and Stchigel.

It is saprobic on dead wood, branches, stems, bark, leaves, freshwater, soil, and sediments of hypersaline lagoons. *Sexual state* is—*Ascomata* usually ostiolate (perithecioid), occasionally non-ostiolate (cleistothecioid), scattered singly or beneath a blackened clypeus, or within a stromatic development of hyphae, peridium mostly thick-walled and composed several layers of flattened cells, occasionally thin-walled (when cleisthotheciod), and very rarely cephalothecoid. *Hamathecium* mostly composed of cellular, branched, septate, numerous pseudoparaphyses, and is occasionally paraphysate. *Asci* mostly 8-spored, uni- to biseriate, bitunicate, clavate to elongate-clavate or cylindrical, sometimes with a minute ocular chamber at the top, wall somewhat thickened, and tapered below or with a somewhat elongated stalk. *Ascospores* rarely unicellular, then globose, mostly multicellular, then ellipsoidal, bi-cupulate, fusoid or fusiform and with one or few transverse septa, medial septum mostly constricted, or muriform, then with one or more longitudinal septa, outer wall smooth-walled, papillate–tuberculate, verruculose, verrucose, or with longitudinal striations, surrounded or not by a mucilaginous sheath, and very rarely with polar appendages. *Asexual state* is — coelomycetous and pycnidial.

A dichotomous key to the accepted species of *Montagnula* is provided as Supplementary Material (S1).

Given that *Montagnula jonesii* was positioned in our phylogenetic analysis distantly from the *Montagnula* spp. main clade and exhibits a phylogenetic distance comparable to one of the coelomycetous genus *Neptunomyces*, we propose reclassifying this fungus into the new genus *Neomontagnula*, as described below.

*Neomontagnula* Barnés-Guirado, Stchigel and Cano, gen. nov. MycoBank MB 854535.

*Etymology*: From Latin neo-, new, because of the phylogenetic and phenotypic relationship with the genus *Montagnula*.

*Description*: *Sexual state*—*Ascomata* solitary, scattered to clustered, immersed or erumpent on the host tissue, globose to subglobose, glabrous, uniloculate, and ostiolate. *Peridium* composed of two layers of pseudoparenchymatous cells, and the outer layer is arranged in a textura angularis. *Pseudoparaphyses* distinctly septate, not constricted at the septa, and anastomosing at the apex. *Asci* 8-spored, bitunicate, fissitunicate, clavate, long pedicellate, and apically rounded, with an ocular chamber. *Ascospores* are overlapping, 1–2-seriate, brown to reddish brown at maturity, transversally 3-septate when mature, fusiform, with rounded ends, constricted at the septa, straight to curved, enlarged at the second cell from the apex, smooth-walled, and guttulated. *Asexual state*—unknown.

*Type species*: *Neomontagnula jonesii* (Tennakoon, Hyde, Wanasinghe, Bahkali, Camporesi, Khan and Phookamsak 2016) Barnés-Guirado, Stchigel and Cano, comb. nov. MycoBank MB 854536.

*Description*: Tennakoon, Hyde, Wanasinghe, Bahkali, Camporesi, Khan, and Phookamsak (2016).

*Notes*: *Neomontagnula jonesii* produces reddish-brown, transversally 3-septate ascospores, such as in *M. aloes*, *M. camporesii*, *M. cirsii*, *M. scabiosae*, and *M. shangrilana*. However, the ascospores of *N. jonesii* are smaller (14–16 × 5–6 μm) than in the previously consigned species of *Montagnula* (33–36 × 13–14 μm in *M. aloes*; 18–25 × 5–8 μm in *M. camporesii*; 18–23.5 × 6.5–9.5 μm in *M. cirsii*; 20–23 × 7–9 μm in *M. scabiosae*; and 48–60 × 17–22 µm in *M. shangrilana*) and have an enlarged second cell from the apex [66,67,68]. *Montagnula aquatica* also produces four-celled ascospores. But, these are dark brown when mature, and their ascomata are globose (lenticular in *N. johnesii*) and smaller (140–210 μm diam. vs. 250–340 × 250–430 μm). *Neomontagnula jonesii* differs from the closest genus *Neptunomyces* by having a sexual state and lacking an asexual coelomycetous state, exactly the opposite of *Neptunomyces* [69].

*Xylariales* Nannf., Nova Acta R. Soc. Scient. upsal., Ser. 4 8 (no. 2): 66 (1932). Mycobank MB 90505.

Diatrypaceae Nitschke [as ’Diatrypeae’], Verh. naturh. Ver. preuss. Rheinl. 26: 73 (1869). Mycobank MB 80692.

*Monosporascus* Pollack and Uecker, Mycologia 66(2): 348 (1974). Mycobank MB 3260.

**Figure 7:**
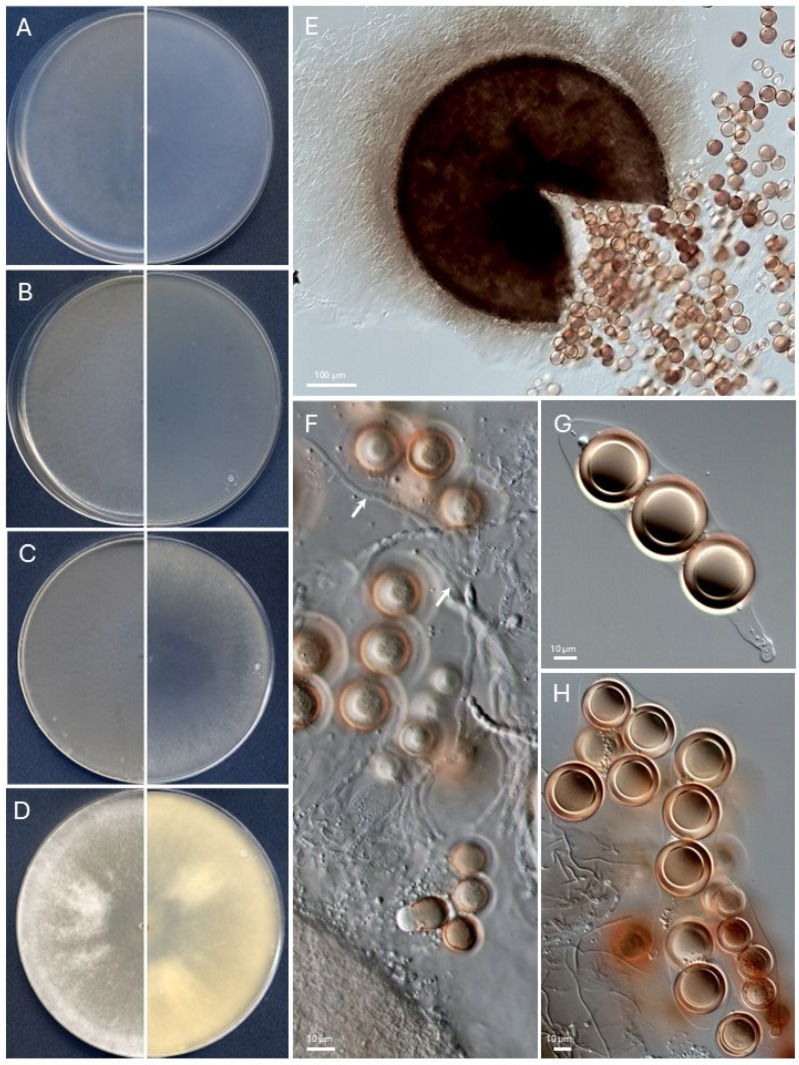
*Monosporascus auratispora* CBS 149967^T^. (**A**–**D**). Colonies on PCA, OA, MEA, and PDA, respectively, after two weeks at 25 ± 1 °C (left, surface; right, reverse). (**E**). Broken ascoma. (**F**). Hymenium (paraphyses, white arrows). (**G**). Mature, 3-spored ascus. (**H**). Asci and free ascospores. Scale bars: (**E**) = 100 µm; (**F**–**H**) = 10 µm.

*Monosporascus auratispora* Barnés-Guirado, Cano and Stchigel, sp. nov. MycoBank. MB 84809 Figure 7.

*Etymology*: From Latin auratus-, golden, -sporae, spores, because of the color of the ascospores under the microscope.

*Description*: *Mycelium* superficial to immersed and composed of hyphae hyaline to pale brown, septate, smooth- and thick-walled, branched, and 1.0 µm wide. *Sexual state* — *Ascomata* immersed to semi-immersed, scattered, pale to dark brown, translucent, non-ostiolate, globose, and 350–550 µm diam. *Peridial wall* subhyaline to brown, translucent, tomentose, 8 to 14-layered, texture epidermoid to angularis, 25–35 µm thick, and consisting of pale yellow to pale brown, thin-walled cells. *Asci* one-to-5-spored (mostly 2–3 spored), fasciculate, short stipitate, clavate to subcylindrical, 35–90 × 15–30 µm, and rounded at the apex, with no apical apparatuses. *Ascospores* are one-celled, hyaline when young, becoming brown at maturity, very thick-walled, three-layered, smooth-walled to slightly granulose, globose, and 20–30 µm diam., without germ pores. *Asexual state* — not observed.

*Culture characteristics* (14 days at 25 °C) Colonies on PDA reach 85 mm diam., flattened, cottony, round, hyaline, and white (1A1) at the center to white (1A1) and hyaline at the margins, filamentous margins, sporulation absent; reverse white (1A1), and soluble pigment absent. Colonies on PCA and OA reach 85 mm diam., flattened, velvety, filamentous, hyaline, sporulation absent; reverse hyaline, and soluble pigment absent. Colonies on MEA reach 85 mm diam., flattened, velvety, filamentous, hyaline at the center, white (1A1) at the margins, sporulation absent; reverse hyaline at the center, white (1A1) at the margins, and soluble pigment absent. The cardinal temperatures of growth are a minimum of 15 °C, an optimum of 30 °C, and a maximum of 37 °C.

*Specimen*: SPAIN, Aragon Community, Zaragoza province, *Salada Grande de Chiprana*, 41°14′19.2″ N 0°10′49.6″ W, isolated from lagoon sediment, 11/07/2022, collected by María Barnés, Alan O. Granados and José F. Cano, isolated by María Barnés, holotype CBS H-25251, and culture ex-type FMR 20333 = CBS 149967.

*Notes*: *Monosporascus auratispora* can be distinguished from its phylogenetically closest species, *Monosporascus ibericus*, by producing smaller ascomata (350–550 µm diam. vs. 400–700 µm diam) and asci (35–90 × 15–30 µm compared to 100–240 × 32–72 µm). Additionally, the asci of *Mo. auratispora* lacks apical structures, whereas those of *Mo. iberic*us exhibit a broad distinct apical ring.

A dichotomous key to the accepted species of the genus *Montagnula* is provided in the Supplementary Material (S2).

## 4. Discussion

The family *Didymosphaeriaceae* was established by Munk in 1953 to accommodate the genus *Didymosphaeria* [70]. Nowadays, there are 33 accepted genera in this family, with most of these reported as saprobes, phytopathogens, and endophytes of a broad diversity of living plants. But, some of them are also pathogens for animals and even humans [67,71]. The genus *Montagnula* is typified by *M. infernalis* (no molecular data available) and was established in 1896 by Berlese [72]. This genus has 47 species according to the Index Fungorum [https://www.indexfungorum.org, accessed on 3 January 2025], but only 22 have available molecular data. Most of the species belonging to this genus are characterized by their sexual state, and up to the description of *M. globospora* in the present article, *M. cylindrospora* was the only species with an asexual (coelomycetous) state [67,73]. Although *Montagnula* species are known to have a global distribution, most species are reportedly associated with a broad diversity of plant hosts from terrestrial non-extremophilic habitats [67]. A recent metabarcoding study [68] revealed that only 1% of the ITS sequences correspond to the *Montagnula* spp. available on the GlobalFungi database (https://globalfungi.com, [accessed on 04 December 2023]) were obtained from aquatic environments, 3% from mangroves, and 1% from wetlands, whereas the biome with the most sequences reported is the forest, with already 61% of total sequences, confirming that this genus is mostly terrestrial and plant-related. Furthermore, this study revealed that less than 2% of the sequences came from extremophilic environments (1% from deserts and 0.1% from tundra) [68]. Our strains FMR 19580 and FMR 20550 were isolated from sediments of two endorheic lagoons, which are aquatic and extremophilic environments. Due to the loss of water by evapotranspiration and percolation throughout the year, endorheic lagoons can suffer dramatic changes in surface extent and salt concentration, with portions of their sediments drying out for several months [1,5]. The strain FMR 19580 exhibits both phenotypic and phylogenetic similarities to three fungal strains isolated in the 1990s by John C. Krug. These strains were recovered from soil samples collected near streams in Egypt (TRTC 51987 = CBS 100254), Malawi (TRTC 51988 = CBS 100256), and Tunisia (TRTC 51989 = CBS 100394), regions characterized by climates comparable to that of the site where strain FMR 19580 was isolated. At first, these strains were assigned to the genus *Zopfia*, but later, they were renamed *Aporospora terricola*, a new fungal taxon. Despite being deposited in several international collections of fungi, no valid taxonomic placement was given for them. However, our study reveals that these strains, along with our strain FMR 19580, belong to the genus *Montagnula* and represent a novel species for that genus, *Montagnula terricola*. *Montagnula terricola* match with certain features of the genus *Montagnula*, such as a peridium with textura angularis, the bitunicate and cylindric-clavate asci, and brown, bicellular, transversely 1-septate, broadly fusiform ascospores [74,75]. However, *M. terricola* differs from most of the species of the genus by producing setose, non-ostiolate, and cleistothecial ascomata. On the other hand, although *Montagnula striatispora* was initially misplaced in the genus *Herpotrichia* (*Melanommataceae*), our study revealed that it morphologically and phylogenetically fits in the genus *Montagnula*. *Montagnula striatispora* shares features with the genus *Montagnula,* such as an erumpent ostiolate ascomata, being globose or pear-shaped, clavate asci and brown, bicellular, transversely 1-septate, and broadly fusiform ascospores, but it differs from the rest of the species in the genus by producing ascospores ornamented with longitudinal ridges, thus becoming a novel combination for the genus [66,74]. Despite our strain FMR 20550 displaying morphological features never reported for species of *Montagnula*, like the cleistothecial ascomata with a cephalothecoid peridium and the unicellular, globose, granulated ascospores, it is placed into the *Montagnula* main clade in our phylogenetic tree and shares other characteristics with other species of the genus, such as the presence of a hamathecium (but composed of paraphyses instead the most common cellular pseudoparaphyses), and the bitunicate, five-to-eight spored, and cylindric-clavate to clavate asci. Consequently, FMR 20550 represents a new species for the genus, *M. globospora* [76]. Moreover, considering the great phylogenetic distance between *M. jonesii* and the *Montagnula* spp. main clade, comparable to that of the *Neptunomyces* spp., we moved *M. jonesii* to the new genus *Neomontagnula*.

The family *Diatrypaceae* was established by Nitschke in 1869, with *Diatrype* designated as the type of genus [77]. Members of *Diatrypaceae* are globally distributed and encompass a diverse range of ecological roles, including saprobic species, endophytes, and significant plant pathogens in both terrestrial and marine biomes [78]. Nowadays, 26 genera are accepted in this family [79]. The genus *Monosporascus* has twelve species accepted according to the Index Fungorum [https://www.indexfungorum.org, accessed on 3 January 2025], and it is characterized by its globose, cleistothecial, or perithecial ascomata (in this case with an ostiolate neck), a paraphysate hamathecium, one-to-six spored clavate-to-saccate asci, and opaque (nearly black), globose ascospores with non-germ pores or slits [80,81]. Species of *Monosporascus* are world-wide important pathogens of Cucurbitaceae, especially for melon and watermelon, but have been reported to infect non-cucurbit plants too [82]. Interestingly, according to the environmental conditions for their isolation and in vitro studies on their physiology, the species of this genus are well-adapted to survive and proliferate in arid or semiarid climates, as well as in alkaline and saline soils [82]. *Monosporascus auratispora*, despite the isolation of *Monosporascus* spp. from salty environments not being uncommon, is the first one isolated from the sediment of a hypersaline lagoon. Phylogenetically, *Mo. auratispora* is closely related to *Mo. ibericus*, which was isolated as an endophyte of an unidentified plant in the Ebro Delta (Tarragona province, Spain) [81]. We believe that these circumstances are not a fortuitous event because, given the proximity to the Mediterranean Sea, the surrounding soils have high salinity. Despite being morphologically related species, *Mo. auratispora* and *Mo. ibericus* are easily distinguishable, due to the absence of an apical ring in the asci of *Mo. auratispora* (present in *Mo. ibericus*) and in the color of the mature ascospores (becoming opaque and nearly black in *Mo. ibericus* and remaining translucent and brown in *Mo. auratispora*). We believe that, despite all three fungi, *M. globospora*, *M. terricola,* and *Mo. auratispora*, behaving as salt-tolerant in in vitro assays and not as halophiles, they are true endemic inhabitants of these ecological niches and not mere passers-by. We base this statement on the fact that all of them produce cleistothecioid ascomata, whose evolutionary appearance is related to the type of habitats from which they came (saline lands) and not to their position in the phylogenetic tree of life, as was suggested for other Ascomycota in previous studies [83,84,85,86].

## 5. Conclusions

Hypersaline endorheic lagoons, such as those examined in this study, represent rare, small, and fragile ecological niches of considerable intrinsic value. Due to their unique characteristics, these ecosystems are highly vulnerable to the effects of global warming and anthropogenic pressures. They have a diverse array of endemic organisms that have evolved to thrive under the extreme physicochemical conditions of these habitats. Thus, the need for their conservation is both capital and urgent. Despite their ecological importance, the fungal biota of hypersaline endorheic lagoons and lakes in Europe remains largely uncharted, primarily due to limited research in these specialized environments. This study addresses this gap by contributing critical insights into the mycobiota of these threatened habitats, including the identification and description of three novel ascomycete species: *Montagnula terricola*, *Montagnula globospora,* and *Monosporascus auratispora*. These species, all of them characterized by the production of cleistothecial ascomata, are specifically adapted to the extreme environmental conditions of these lagoons.

## Figures and Tables

**Table 2:** Taxa and GenBank accession numbers of the molecular markers used in the phylogenetic analysis.

Taxon	Strain Number	GenBank Accession Number			
		ITS	LSU	*TEF*-1α	*tub*2
*Alloconiothyrium camelliae*	NTUCC 17-032-1^T^*	MT112294	MT071270	MT232967	
*Alloconiothyrium aptrootii*	CBS 980.95^T^	JX496121	JX496234		
*Austropleospora osteospermi*	BRIP51628^T^	FJ481946			
*Chromolaenicola nanensis*	MFLUCC 17-1473^T^	MN325015	MN325003		
*Chromolaenicola thailandensis*	MFLUCC 17-1510^T^	MN325018	MN325006	MN335651	
*Cylindroaseptospora leucaenae*	MFLUCC 17-2424^T^	NR_163333	NG_066310	MK360047	
*Didymosphaeria rubi-ulmifolii*	MFLUCC 14-0023^T^		KJ436586		
*Eutypa camelliae*	HKAS 107022^T^	NR_175674	NG_081500		
*Eutypa petrakii*	CBS 285.87	MH862077	MH873766		
*Kalmusia ebuli*	CBS 123120^T^	KF796674	NG_070920		
*Kalmusia longispora*	CBS 582.83^T^	MH861658	NG_070449		
*Kalmusibambusa triseptata*	MFLUCC 13-0232	KY682697	KY682695		
*Karstenula lancangensis*	KUMCC 21-0670^T^	OP058969	OP059060		
*Karstenula rhodostoma*	CBS 691.94	LC014559	AB807531	AB808506	
*Monosporascus auratispora* sp. nov.	**FMR 20333^T^**	**PP973385**	**PP973719**	**PP973931**	**PP973383**
*Monosporascus brasiliensis*	CMM-4839^T^	MG735234	MG748803	MG720040	MG725317
	CMM-4840	MG735235	MG748804	MG720041	MG725318
*Monosporascus bulgaricus*	CBS 151406^T^	KT269184	PP454707		PP460994
	P1811	KT269083			
*Monosporascus caatinguensis*	CMM-4833^T^	MG735228	MG748797	MG720034	MG725311
	CMM-4834	MG735229	MG748798	MG720035	MG725312
*Monosporascus cannonballus*	CBS 609.92^T^	NR_111370			
	MC0603	JQ762364	MG748824	JQ907314	JQ907307
*Monosporascus europaeus*	CBS 150022^T^	KT269082	PP454705		PP481183
	P1889	KT269158	PP454706		
*Monosporascus eutypoides*	MT45^T^	JQ958963	MG748827	JQ958959	JQ973834
	MT47	JQ958964		JQ958956	
*Monosporascus ibericus*	CBS 110550^T^	JQ973832	JQ958958	JQ958958	JQ973833
*Monosporascus mossoroensis*	CMM-4857^T^	MG735252	MG720058	MG720058	MG725335
	CMM-4858	MG735253	MG720059	MG720059	MG725336
*Monosporascus nordestinus*	CMM-4846^T^	MG735241	MG720047	MG720047	MG725324
	CMM-4847	MG735242	MG720048	MG720048	MG725325
*Monosporascus solitarius*	CBS 150023^T^	KT269777	PP454708		
*Monosporascus semiaridus*	CMM-4830^T^	MG735222	MG748791	MG720028	MG725305
	CMM-4831	MG735223	MG748792	MG720029	MG725306
*Montagnula acaciae*	MFLUCC 18-1636	ON117280	ON117298		
	NCYUCC 19-0087^T^	ON117281	ON117299		
*Montagnula aloes*	CBS 132531^T^	NR_111757	NG_042676		
*Montagnula appendiculata*	CBS 109027^T^	DQ435529	AY772016		
*Montagnula aquatica*	MFLU 22-0171^T^	OP605992	OP605986		
	KUNCC 23-14425	OR583097	OR583116	OR588088	
*Montagnula aquilariae*	KUNCC 22-10815^T^	OP452927	OP482265	OP426318	
	KUNCC 22-10816	OP554219	OP482266	OP426319	
*Montagnula bellevaliae*	MFLUCC 14-0924^T^	KT443906	KT443902		
*Montagnula camporesii*	MFLUCC 16-1369^T^	MN401746	NG_070946	MN397908	
*Montagnula chiangraiensis*	MFLUCC 17-1420^T^	NR_168864	NG_068707		
*Montagnula chromolaenae*	MFLUCC 17-1435^T^	NR_168865	NG_068708		
*Montagnula cirsii*	MFLUCC 13-0680	KX274242	KX274249	KX284707	
*Montagnula cylindrospora*	CBS 146572^T^	LT796834	LN907351	LT797074	
*Montagnula donacina*	HVVV01	KJ628375	KJ628377		
	MFLUCC 17-1469	NR_168866	NG_070948	MT235773	
*Montagnula graminícola*	MFLUCC 13-0352^T^	KM658314	KM658315		
*Montagnula guiyangensis*	HKAS 124556^T^	OP605989	OP600484		
	GUCC 22–0817	OP605990	OP600485		
*Montagnula krabiensis*	MFLUCC 16-0250^T^	NR168179	NG068826	MH412776	
*Montagnula lijiangensis*	HKAS 126540	OR583107	OR583126	OR588098	
	HKAS 126541^T^	OR583108	OR583127	OR588099	
*Montagnula menglaensis*	KUNCC 23-14424^T^	OR583111	OR583130	OR588102	
	KUNCC 23-14423	OR583110	OR583129	OR588101	
*Montagnula opulenta*	CBS 168.34	DQ678086	DQ678086		
*Montagnula scabiosae*	MFLUCC 14-0954^T^	NR_155378	NG_059602		
*Montagnula shangrilana*	KUNCC 23-14433	OR583112	OR583131	OR588103	
	KUNCC 23-14434^T^	OR583113	OR583132	OR588104	
*Montagnula striatispora*	CBS 385.65^T^	MH858624	NG_064048		
*Montagnula terricola* sp.nov.	FMR 19580	**OQ708962**	**PP973381**	**PP973930**	
	CBS 100254	**OQ708960**	**PP973379**	**PP973928**	
	CBS 100256^T^	**OQ708961**	**PP973380**	**PP973929**	
	CBS 100394	**OQ708959**	**PP973378**	**PP973927**	
*Montagnula thevetiae*	HKAS 126963	OR583114	OR583133	OR588105	
	HKAS 126964^T^	OR583115	OR583134	OR588106	
*Montagnula globospora* sp.nov.	FMR 20550^T^	PP973384	PP973382	PP973931	
*Montagnula verniciae*	CGMCC 3.24435^T^	OR269139	OR253273	OR251166	
	UESTCC 23.0029	OR269140	OR253274	OR251167	
*Neokalmusia brevispora*	CBS 120248	MH863078	MH874633		
*Neokalmusia jonahhulmei*	KUMCC 21-0818^T^	ON007043	ON007039	ON009133	
*Neomontagnula jonesii* gen. nov.	MFLUCC 16-1448^T^	KY313619	KY273276	KY313620	
	MFLU 18-0084	ON117282	ON117300	ON158095	
*Neptunomyces aureus*	CMG10A^T^	MK912119		MK947998	
	CMG13	MK912122		MK948001	
*Neptunomyces litoralis*	BRIP 75555a^T^	OR271911	NG_242141		
*Paracamarosporium psoraleae*	CPC 21632^T^	KF777143	KF777199		
*Paracamarosporium hawaiiense*	CBS 120025^T^	NR_154287	NG_070608		
*Paraconiothyrium estuarinum*	CBS 109850^T^	NR_166007	MH874432		
*Paramassariosphaeria anthostomoides*	CBS 615.86	MH862005	MH873693		
*Paramassariosphaeria clematidicola*	MFLUCC 16-0172	KU743206	KU743207		
*Paraphaeosphaeria michotii*	CBS 591.73	MH860778	GU456326	GU456267	
*Paraphaeosphaeria rosae*	MFLUCC 17-2547^T^	MG828935	MG829044	MG829222	
*Periconia didymosporum*	MFLU 15-0057^T^	NR_176693	NG_081448	KP761727	
*Periconia alishanica*	MFLUCC 19-0145^T^	MW063165	MW063229	MW183790	
*Pseudocamarosporium piceae*	MFLUCC 14-0192^T^	KJ747046	KJ803030		
*Pseudocamarosporium propinquum*	MFLUCC 13-0544^T^	NR_154309	NG_069202		
*Septofusispora thailandica*	MFLU 22-0043^T^	OP058971	OP059062	OP135945	
*Verrucoconiothyrium nitidae*	CBS 119209^T^	EU552112	EU552112		
*Xenocamarosporium acaciae*	CBS 139895^T^	NR_137982	NG_058163		

^T^* = Ex-type strains. FMR: Facultad de Medicina y Ciencias de la Salud, Reus, Spain; CBS: culture collection of the Westerdijk Fungal Biodiversity Institute, Utrecht, The Netherlands; MFLUCC: culture collection of the Mae Fah Luang University, Chiang Rai, Thailand; MFLU: herbarium of the Mae Fah Luang University, Chiang Rai, Thailand; NTUCC: National Taiwan University Culture Collection, Taipei. En Taiwan; BRIP: The Plant Pathology Herbarium, Queensland, Australia; HKAS: herbarium of the Kunming Institute of Botany, Kunming, China; KUMCC/KUNCC: Kunming Institute of Botany Culture Collection Kunming, China; CMM: culture collection of phytopathogenic fungi Prof. Maria Menezes, Dois Irmaos, Brazil; NCYUCC: National Chiayi University Culture Collection, Chiayi City, Taiwan; HVVV: personal collection of Wayne Pitt from Vitis vinifera, Bathurst, Australia; GUCC: culture collection at Department of Plant Pathology, Agriculture College, Guizhou University, Guizhou, China; CGMCC: China General Microbiological Culture Collection Center, Beijing, China; UESTCC: University of Electronic Science and Technology Culture Collection, Chengdu, China; CMG: culture collection of Mark Gleason, Ames, USA; CPC: culture collection of P.W. Crous, Utrecht, The Netherlands. In bold, sequences generated in this study.

**Table 3:** BLAST results for our strains FMR 19580, FMR 20550, and FMR 20333.

Strain	Molecular Marker	Closest Species	Identity (%)	GenBank Accession Number	Identities
FMR 19580	ITS	*Aporospora terricola* *	99.78%	AF049088.1	446/447 (no gaps)
		*Herpotrichia striatispora* CBS 385.65	98.43%	MH858624.1	440/447 (no gaps)
	LSU	*Herpotrichia striatispora* CBS 385.65	99.50%	NG_064048.1	796/800 (no gaps)
		*Montagnula cylindrospora* UTHSC DI16-208	98.75%	LN907351.1	790/800 (4 gaps)
		*Montagnula bellevaliae* MFLUCC 14-0924	98.50%	NG_059601.1	788/800 (2 gaps)
	*TEF*-1α	*Montagnula donacina* MFLU 22-0046	95.91%	OP135938.1	820/855 (no gaps)
		*Montagnula puerensis* KUMCC:20-0225	95.49%	MW573959.1	763/799 (no gaps)
FMR 20550	ITS	*Herpotrichia striatispora* CBS 385.65	96.42%	MH858624.1	431/447 (7 gaps)
		*Montagnula terricola* FMR 19580	96.42%	OQ708962.1	431/447 (7 gaps)
	LSU	*Herpotrichia striatispora* CBS 385.65	98.26%	NG_064048.1	791/805 (6 gaps)
		*Montagnula cirsii* MFLUCC:13-0680	97.86%	KX274249.1	787/804 (6 gaps)
	*TEF*-1α	*Montagnula donacina* MFLUCC:22-0046	96.23%	OP135938.1	842/875 (no gaps)
		*Montagnula chromolaenicola* MFLUCC:17-1469	96.03%	MT235773	846/881 (no gaps)
FMR 20333	ITS	*Monosporascus ibericus* CBS 110550	96.60%	JQ973832	454/470 (5 gaps)
	LSU	*Monosporascus caatinguensis* CMM-4832	97.37%	MG748796	667/685 (5 gaps)
		*Monosporascus eutypoides* CBS:132472	96.93%	MH877468	664/685 (8 gaps)
	*tub2* *TEF*-1α	*Monosporascus ibericus* CBS 110550	98.62%	JQ973833	502/509 (1 gap)
		*Monosporascus ibericus* CBS 110550	93.27%	JQ958958	554/594 (6 gaps)

* non-legitimate name; author’s note.

## Data Availability

Data are contained within the article.

## Supplementary Materials

The following supporting information can be downloaded at https://www.mdpi.com/article/10.3390/jof11020139/s1: S1: Dichotomous key of accepted species of *Montagnula* (adapted from Sun, Y.-R.; Zhang, J.-Y.; Hyde, K.D.; Wang, Y.; Jayawardena, R.S. Morphology and phylogeny reveal three *Montagnula* species from China and Thailand. Plants 2023, 12, 738. https://doi.org/10.3390/plants12040738 [67]); and S2: Dichotomous key of the species of *Monosporascus*.

## Abbreviations

The following abbreviations are used in this manuscript:

AA	Ascospore Agar
BI	Bayesian Inference
BLAST	Basic Local Alignment Search Tool
BRIP	The Plant Pathology Herbarium
BS	Bootstrap Support
CBS	Westerdijk Fungal Biodiversity Institute
CGMCC	China General Microbiological Culture Collection Center
CMG	Culture collection of Mark Gleason
CMM	Culture Collection of Phytopathogenic Fungi Prof. Maria Menezes
CPC	Culture Collection of P.W. Crous
FMR	Faculty of Medicine of Reus
G18	18% of Glycerol Agar
GUCC	Culture Collection at Department of Plant Pathology, Agriculture College, Guizhou University
HKAS	Herbarium of the Kunming Institute of Botany
HVVV	Personal collection of Wayne Pitt from Vitis vinifera
ITS	Internal Transcribed Spacers
KUMCC/KUNCC	Kunming Institute of Botany Culture Collection Kunming
LSU	D1-D2 domains of the 28S nrRNA
M.	Montagnula
MEA	2% Malt Extract Agar
MEGA	Molecular Evolutionary Genetics Analysis
MFLU	Herbarium of the Mae Fah Luang University
MFLUCC	Culture Collection of the Mae Fah Luang University
ML	Maximum Likelihood
MLI	Maximum Level of Identity
Mo.	Monosporascus
NCY-UCC	National Chiayi University Culture Collection
NTUCC	National Taiwan University Culture Collection
OA	Oatmeal Agar
PCA	Potato–Carrot Agar
PDA	Potato–Dextrose Agar
PP	Posterior Probability
TEF-1α	Fragments of the Translation Elongation Factor 1α
tub2	Beta-tubulin gene
UESTCC	University of Electronic Science and Technology Culture Collection
