# Supplementary material for: New Species of Ascomycetes from Two Hypersaline Endorheic Lagoon Complexes in Zaragoza Province (Aragon Community, Spain)
Source: J Fungi (Basel). 2025 Feb 12;11(2):139. doi: 10.3390/jof11020139 (PMC11856669; doi:10.3390/jof11020139)
Supplement: Supplementary file 1 [file jof-11-00139-s001.zip › jof-3468663-supplementary.pdf]

S1. *Dichotomous* key of accepted species of *Montagnula* (adapted from Sun, Y.-R.; Zhang, J.-Y.; Hyde, K.D.; Wang, Y.; Jayawardena, R.S. Morphology and phylogeny reveal three *Montagnula* species from China and Thailand. *Plants* 2023, 12, 738. https://doi.org/10.3390/plants12040738).

1. Ascomata non-ostiolate or cleistothecial 2
1’. Ascomata ostiolate 5

2. Ascomata non-ostiolate produced in the host..3
2’. Ascomata cleistothecial produced *in vitro*..4

3. Coelomycetous (pycnidial) asexual state formed *in vitro*; ascospores bicellular, biconical, 10.5–14 × 4.5–5. µm, upper cell wider than lower cell, surrounded by a sheath thickened at both ends ....*M. menglaensis*
3’. Asexual state unknown; ascospores bicellular, fusiform, 25–32 × 6–7 µm, upper and lower cells similar in size and shape, surrounded by a regular sheath……………………………………………………………..*M. krabiensis*

4. Peridium cephalothecoid; ascospores unicellular, globose, superficially granulate; coelomycetous asexual state formed in culture ....*M. globospora*
4’. Peridium non-cephalotecoid; ascospores bicellular, bi-cupulate, symmetrical……………………………………………………..*M. terricola*

5. Ascospores are didymosporous .... …6
5’. Ascospores are phragmosporous... ..17
5’’. Ascospores are dictyosporous.. ..23

6. Didymospores sheathed.. ...7
6’. Didymospores without sheath.. ..14

7. Didymospores surrounded by a mucilaginous sheath .... …8
7’. Sheath was drawn out to form polar appendages 12

8. Ascospores fusiform, 30–40 × 11.5–14 µm, verruculose ....…*M. thevetiae*
8’. Ascospores elipsoidal.. …9

9. Ascospores asymmetrical, 9–14 × 4–6.1 µm………………..……....*M. vakrabeejae*
9’. Ascospores symmetrical... ..10

10. Ascospores 15–16.5 × 5–6 µm, with prominent appendages…*M. chromolaenae*
10’. Ascospores not appendaged... ..11

11. Ascospores mid brown, 7.8–15 × 2.8–6.5 µm, slightly constricted at the septum……………………………………………………………*M. graminicola*
11’. Ascospores dark brown, 14–18 × 4.5–6 µm, not constricted at the septum……………………………………………………………….*M. palmacea*

11’’. Ascospores dark brown, 22–26 × 10–14 μm, constricted at the septum……………………………………………………………*M. lijiangensis*

12. Ascospores 1-seriate, yellowish brown to brown, 12–15 × 4–5 μm…………………………………………………………..*M. appendiculata*
12’. Ascospores 2–3-seriate, with polar appendages 13

13. Ascomata 300–400 × 350–400 μm; asci 84–135 × 10–15 μm; ascospores 10–20 × 3.5–6 μm…..………………………………………………*M. guiyangensis*
13’. Ascomata 150–220 × 200–230 μm; asci 60–75 × 8–11 μm; ascospores 11–15 × 4–6 μm…………………………..*M. chiangraiensis*

14. Ascomata superficial; ascospores 16–18 × 6–7.5 μm…………..……*M. longipes*
14’. Ascomata immersed or erumpent.. ..15

15. Ascomata 140–180 × 150–200 μm; ascospores 10–15 × 4–7 μm……….*M. acacia*
15’. Ascomata greater than 200 μm 16

16. Ascospores brown, 12–17 × 4–6.5 μm…………………………………..*M. donacina*
16’ Ascospores pale brown, 19–25 × 9–13 μm………………………..……*M. opulenta*

17. Ascomata superficial; ascospores 18–25 × 5–8 μm…………………*M. camporesii*
17’. Ascomata immersed or erumpent... ..18

18. Asci with short stalks 19
18’. Asci with long pedicellate... ..20

19. Ascospores 5 transverse septa, 21–25 × 5–7 μm……………….*M. subsuperficialis*
19’. Ascospores 3 transverse septa, 24–35 × 7.5–14 μm…………..……..*M. aquatica*
19’’. Ascospores 3 transverse septa, 48–60 × 17–22 μm……..............*M. shangrilana*

20. Ascospores with 2 transverse septa, 15-18 × 5-6 μm……..………*M. bellevaliae*
20’ Ascospores with 3 transverse septa 21

21. Ascospores greater than 30 μm, ovoid to ellipsoid………………………...*M. aloes*
21’. Ascospores not more than 30 μm, ellipsoid to fusiform 22

22. Ascomata 385–415 × 510–525 μm, asci 85–120 × 10.5–13.5 μm…………*M. cirsii*
22’. Ascomata 300–320 × 300–360 μm, asci 110–130 × 14–20 μm….......*M. scabiosae*

23. Ascospores with more than 10 transverse septa.. ..24
23’. Ascospores with less than 10 transverse septa.. ..26

24. Ascospores with more than 15 transverse septa,
ascospores 40–45 × 15–17 μm……………………………….....*M. gigantea*
24’. Ascospores with ≤15 transverse septa 25

25. Ascospores 32–40 × 8–9.8 μm, asci 80–110 × 13–15 μm…………………....*M. dura*

25’. Ascospores 31–45 × 13.5–16.5 µm, asci 110–160 × 13–6 µm…..………..*M. triseti*

26. Ascospore transverse septa up to 5 27
26’. Ascospore transverse more than 5 30

27. Ascospores without sheath... ..28
27’. Ascospores with sheath 29

28. Ascospores 2–3 transverse septa, 0–1 longitudinal septum, 12.5–16.5 × 4.8–6.5 µm…………………………………..*M. baatanensis*
28’. Ascospores 5 transverse septa, 1 longitudinal septum, 24–29 × 9–11 µm…………………………………………………………………………*M. infernalis*

29. Ascospores 17.5–23 × 5.5–8.5 µm, fusiform to somewhat broadly fusiform……………………………………………………………………*M. opuntiae*
29’. Ascospores 16–18 × 6–7.5 µm, ellipsoid fusoid……………...……*M. thuemeniana*

30. Ascospores without sheath... ..31
30’. Ascospores with sheath 32

31. Ascospores 17–25 × 7.5–10 µm, 5–7 transverse septa…………………......*M. obtusa*
31’. Ascospores 39–47 × 15–19 µm, 7–9 transverse septa………...………*M. opaca*

32. Ascospores broadly ellipsoid, 5–7 transverse septa……………*M. phragmospora*
32’. Ascospores obovoid fusoid, 7–10 transverse septa 33

33. Ascospores 2–3 longitudinal septa, 40.8–52 × 17.6–22.4 µm……..*M. mohavensis*
33’. Ascospores 1–2 longitudinal septa 34

34. Ascospores 35–50 × 16–20 µm, asci 2–8-spored…………………...*M. dasylirionis*
34’. Ascospores 27–42 × 12–15 µm, asci 4–8-spored………………........*M. yuccigena*

S2. *Dichotomous* key of the species of *Monosporascus*.

1. Ascomata produced……………………………………………………………..........2
1'. Mycelium remaining sterile………………………………………………………...*

2. Ascomata perithecial…………………………………………………………………3
2'. Ascomata non-ostiolate or cleistothecial……………………………………………...4

3. Asci 1(–2)-spored; ascospores 25–55 µm diam. opaque..………..*Mo. cannonballus*
3'. Asci 1–3(–4)-spored; ascospores 21–35 µm diam., opaque…..........*Mo. eutypoides*

4. Peridium non-cephalothecoid………………………………..……………………...5
4'. Peridium cephalothecoid……………………………………………………...........7

5. Asci strictly 1-spored; ascospores 34–52 µm diam., opaque………..*Mo. semiaridus*
5'. Asci distinct..………………………………………………………………………6

6. Asci 1–3-spored; ascospores 35–50 µm diam., opaque..…………..*Mo. nordestinum*
6'. Asci 1–6-spored; ascospores 18–60 µm diam., opaque..…………..……..*Mo. ibericus*

7. Asci strictly 1-spored; ascospores reticulate, opaque………………*Mo. monosporus*
7'. Asci 1–5-spored; ascospores smooth-walled, never opaque………*Mo. auratispora*

* Includes *Mo. brasiliensis*, *Mo. bulgaricus*, *Mo. caatinguensis*, *Mo. europaeus*, *Mo. mossoroensis* and *Mo. solitarius*.
